# Operative Dentistry

**Published:** 1892-11

**Authors:** B. F. Arrington

**Affiliations:** Asheville, N. C.


ARTICLE V.

### OPERATIVE DENTISTRY.

BY B. F. ARRINGTON, M. D., D. D. S., ASHEVILLE, N. C.

The first consideration in the care and preservation of children's teeth should be thorough and systematic cleanliness of the teeth. Inspection on the part of mother or nurse should be frequent. Tooth brushes suitable in size, shape and texture of bristles should be freely used several times daily, and always just before retiring at night so as to free the teeth of any impacted or accumulated substances. The mother or nurse should manipulate the brush as in their judgment may be best until children are old enough to reason on the subject and fully comprehend the importance of the operation. All teeth back of the eye teeth should be carefully brushed on the cutting or grinding surface. With such care, the first set of temporary teeth would be retained with freedom from early and extreme decay and be preserved in a comparative state of comfort until discarded by natural process for the inception of permanent teeth. In the event of perceptible decay, with proper application of appropriate instruments and appliances, the defects can be easily and effectually removed and is far better for the teeth and more comfortable to patients of tender years than would be the operation of excavating and filling. If neglected until decay is too far advanced to be removed by means above advised, shape cavities to best advantage possible and fill with gutta-percha, and add to when necessary, or in large grinding surface cavities in molars or bicusps, cap guttapercha with amalgam, and always with an amalgam possessing a large percentage of tin. Cavities in temporary teeth should never be filled with gold, it would be contrary to right, never justifiable would be a very great injustice to children and parents. To preserve the temporary teeth in tact for utility and proper development of maxillaries and space for permanent set of teeth should be the aim of every dentist regardless of any special material to be used or broad margins for profits. Young children should be dealt with in dental offices persuasively, gently and pleasantly, and under no circumstances should one be deceived by an operator.

In regard to extraction of temporary teeth, I will venture the assertion that if the above treatment could and should be successfully conducted, there would be but little or no necessity for extraction. If decay has progressed until there is pulp exposure and pain in consequence, then devitalize nerve and treat as for an adult under similar circumstances.

Arsenic is my favorite remedy for devitalizing nerve.

I have tried pretty much all remedies recommended and find nothing equal to or half so sure of good results as arsenic, the 30th or 40th of a grain is quite sufficient if rightly applied. My mode of application is to moisten a small pellet of cotton with creosote or campho-phenique held with pliers touch to arsenic for the quantity requisite and convey to cavity, carefully guarding against placing in direct contact with pulp, protect against moisture with beeswax or cotton and sandarch, with children the cotton and sandarach is preferable, let remain ten or twelve hours and remove, then after a lapse of six or eight days sufficient time for partial dissolution of pulp and nerve filaments, extirpate, and if successfully, without delay insert filling, first wiping out pulp chamber and roots with campho-phenique. Retain temporary teeth if possible until by natural process of waste they are of no further use. Of course there are some circumstances that will justify and require removal, but spare the forcep and elevator when possible to do so without detriment.

The six year molars, what of them? Should effort be made to preserve them or let them go without much consideration? The procedure as to preservation of the six year molars I consider altogether as circumstancial, thus, if the general contour and development of the maxillaries are delicate and contracted and the teeth large as is sometimes the case, requiring much space, and the delay is far advanced, I would say extract without delay that the next molar may move forward and fill space, thereby making more room for the wisdom teeth, which often prove troublesome, defective and worthless for want of room. If the maxillaries are well developed and promise ample space for all the teeth, then by all means save as best we can the six year molars. The six year molars and wisdom teeth are often as durable as any other teeth in the mouth, all things being equal. Regular systematic mastication on the six year molars as soon as erupted and continued, and the tooth brush rightly applied with persistent regularity would insure usefulness and freedom from decay as certainly as any teeth in the mouth similarly treated, wisdom teeth likewise when not too much crowded. Any tooth or teeth to be healthy and preserved in a comparatively normal state, must be freely and regularly used, and sometimes, I might say often, the harder the substance masticated in reason, the better for health of teeth and gums.

Treating decay and filling teeth, I hold that any decay that can be successfully removed without detriment to the pulp, leaving the space smoothly polished and self cleansing, is wiser and better practice than to excavate and fill. Free separations and appropriate conservative treatment of defective surface is less expensive, less hurtful and harmful than filling. Decay around the necks of teeth, bucal and labial can be treated and the teeth better preserved by properly shaping, medicating and dressing, than by filling. I seldom ever fill that class of decay, having learned from experience long since that the use of chisels, finishing burs, corundum points, direct application of Nit. silver, creosote, Sul. acid, &c., followed with stick and polishing powders, will accomplish more for the comfort of my patients and preservations of their teeth than excavating burs, excavators and pluggers, with great saving of time and labor to operator and money to patients. Another very important consideration in favor of this line of practice, is the freedom from glaring exposure of gold as is often seen in mouths, and is always unsightly and often very objectionable, and if possible to be avoided, should never be tolerated in practice. I have examined the condition of teeth treated as above advised six or eight years after treatment and found them entirely satisfactory; no recurrence of decay and entire freedom from sensation as much so as any portion of crown in a normal state. Could a better result be desired, or obtained by excavating and filling with gold, the most popular material for such cavities, or any other material? I do not only question it, but unhesitatingly assert without fear of contradiction based upon practice and experience, that so good a result could not be obtained. If the decay has advanced so far as to require excavating and filling, the question then arises as to material best suited, all things considered. Under some circumstances, gold is admissible and should be used, but in a large majority of cases guttapercha is preferable, it will serve a better purpose than any other material. I have patrons for whom I have inserted guttapercha fillings in labial and bucal cavities five and six years ago and the fillings are now in a good condition and bid fair to give satisfaction for years to come.

When I first commenced the use of guttapercha in such cavities, I feared the waste from friction in use of brush would be rapid and prove a hindrance to use of the article, or necessitate the operations of frequent adding to, but time and careful observation has convinced me to the contrary. The secret of success in the use of guttapercha consists in proper introduction and thorough condensation of material from base to surface. Never crowd or stuff a cavity with large pieces and never apply too much heat, better to apply heated instruments to material than material to heat. Every undercut and irregularity of cavity surface should be considered and worked properly, and special care to condensing at cervical section of cavities, especially in approximal cavities. No metal except cohesive tin can be so condensed into a cavity as to insure against leakage, as a good and reliable article of guttapercha, results justify the assertion, though greatly at variance with the expressed opinion of some of our most experienced and best operators. Facts established through practical experience and careful observation for results, and the results satisfactory will do to assert and defy contradiction. It is my honest judgment and conviction from experience that more teeth from early childhood to old age can be preserved in comfort and for usefulness with guttapercha than with gold singly or any other material now known to the profession, and it is only a question of time when guttapercha or some corresponding article will be in the lead of all material for filling cavities in teeth, and dentistry will not be on the decline, but the advance onward and upward.

When it was asserted and advocated a quarter of a century ago that rubber would take the place of gold as a base for artificial dentures, many protested and there went up a howl against it from Main to Mexico, and some gold advocates were so extreme in their prejudices and tied to their one idol gold, as to refuse to receive and impart instruction to dental students, unless they would pledge themselves not to use rubber in their practice. An extreme feature of contracted prejudice reduced to a point, censurable and pitiable. In the face of such opposition, rubber prevailed as was right it should. Thousands of the human family have been blessed with the privilege and use of artificial dentures on rubber-base at reasonable living rates, who must have suffered for want of them had the gold theory prevailed. The profession has rapidly advanced and gold quietly retired to the rear, to serve only in the future as emergency requires. So it may be, doubtless will be with gold as a filling material, in relation to guttapercha and similar preparations in operative dentistry short of the next quarter of a century. Humanity and true science freed of prejudice must and will prevail, and to the great credit of our profession.

Amalgams, especially those composed largely of tin serves a good purpose as a filling material and in a large majority of cavities will prove more durable than gold, and with the majority of persons in need of fillings as entirely satisfactory as gold, and for all classes would be as satisfactory, except as to appearance. Those who have through weakness, a fancy for display of gold in or on their teeth must be accommodated I suppose to some extent, possibly for a decade or so longer.

Some people are just so constituted, vain, weak and wanting in judgment, they will insist upon the use of gold for filling, even if definitely informed, and positively certain the result would be unfavorable and teeth would not last so long as if filled with some other material. A weakness inexcusable, and must be regulated and corrected in course of time through education and a right advocacy of the right in all things pertaining to dentistry on the part of operators in the discharge of professional duties in their respective offices.

The weakness for display of gold has increased surprisingly during the past ten or fifteen years. Some people when consulting concerning treatment of their teeth when informed the chances are favorable for successful preservation by filling, will reason and argue thus: I will not have them filled now, they will probably last longer as they are without giving trouble, I will then have them crowned with gold. Any dentist who would sanction such a practice and not bodly advise against it is unworthy of the name of dentist. I am sorry to say I fear there are some such.

The growing disposition on the part of operators to display gold in the mouth (often as an advertisement) is an unfavorable and demoralizing feature in practice, and a crying shame upon a noble and useful profession. Gold is good and commendable as a filling material; it has served a good purpose and the use of it in filling teeth will be continued, and he or she who manipulates it skillfully and effectively deserves credit, but it is not the only material of merit for filling as some claim. It often serves a good purpose and under some circumstances can be advantageously used and in certain cases is superior to any other material, but to attempt to force its use in filling all cavities and under all circumstances is an absurdity and proof evident that those who so advocate are contracted in their ideas and limited in knowledge as to the best means of treating and preserving the natural teeth, and it is through such advocacy of gold fillings and ignorance of the more conservative and broader scope of treatment requisite and practiced for the preservation of teeth that caused the wholesale extraction of teeth as now practiced and creates demand for artificial teeth. Less prejudice and more liberal minded conservatism in practice would be a wiser course, and the human family would thereby be profited. Only a short while ago, I read in a Dental Journal a declaration from an aged prominent member of the profession, and a professor in a dental college, that he had never filled a cavity with amalgam, inexcusable prejudice, and non-progressive, such a man should vacate position as teacher in a dental college and let some one less prejudiced and more progressive take his place. Conservatism and progress must be respected and encouraged. New remedies, material, instruments and appliances must be tested (rightly and thoroughly tested) and when found to possess true worth and can be used to advantage, give them a place as they merit, and in the interest of humanity and our profession, endorse and commend fearlessly and freely that the profession may advance and become truly progressive, plainly perceptible at the close of every decade, any action short of this will prove hurtful and a hindrance to professional elevation. Amalgam is daily growing in favor, prejudice abating with same, there is less abuse in the use of the article, in most of the colleges; students are instructed in the use of amalgam as in the use of amalgam as in the use of gold and other material, an encouraging feature, truly right and proper. A better class of work is being done and better results obtained than in former years. I am inclined to the opinion and will predict that twenty years hence, ninety per cent. of teeth filled in this country will be with plastic material, and a larger per cent. of teeth preserved than if gold alone or any one metal should be used. Time will determine the correctness of the prediction.

Under many circumstances guttapercha must be combined with amalgam for successful preservation of teeth by filling. In deep crown, approximal and bucal cavities, the base should be thoroughly covered with guttapercha and carefully condensed until cavity is one-fourth or one-third filled, a perfect safe-guard against injurious effects upon the nerve pulp from heat or cold. Then fill to completion with amalgam. Approximal cavities are the cavities of all cavities that require special care in preparation for filling, and manipulation of material in filling, and after all care and pains-taking defects will present sooner than in any other class of cavities, matters not with what filled. In the use of gold or amalgam it is admissable and advisable to pack the cervical section of cavity with guttapercha, a few years experience will satisfy anyone of the value of guttapercha thus applied. Whenever there is decay around an amalgam or gold filling at cervical section as is quite often seen around fillings as perfect as when first inserted, it is unwise and bad practice to repair with same material, guttapercha is preferable, it can be more easily applied and packed in cavity, and will better preserve against decay. Tin foil used in cylinder form, packed at cervical section of cavities to be filled with gold is good practice, preserves a tooth longer than if filled with all gold. A preparation of tin known as cohesive tin prepared by the S. S. White Dental Manufacturing Company, is an excellent article for filling children's teeth, it can be easily and rapidly introduced and condensed, makes a good water-tight filling and lasts very well, may be placed in close approximation to nerve pulp and cause no discomfort. It is an admirable adjunct to gold in filling deep cavities for adults. Fill cavity half or three-fourths full of cohesive tin and finish with gold, is far better for comfort and preservation of teeth than to fill with gold from base to completion. Many teeth can be filled and made useful for years with cement when nothing else could be used to advantage, and preserve so well the natural appearance of the teeth. I make free use of it and am pleased with results and so are my patrons. In the use of cements, it is all important to preclude moisture during the operation of filling, and for an hour or so afterwards, which can be easily done by a right application of beeswax. All cements prove more durable in crown cavities than approximal, bucal or labial, and why? I would like to know. Experience teaches that the more open the approximal space the more durable the filling, not only cement fillings, but gold, amalgam and tin likewise.

Whenever approximal cavities are filled with metal, the space between the teeth should be left sufficiently open to admit of the free use of quill tooth picks, bristles of brush, linen tape, etc., but for floss silk never, no good but sometimes much harm is done by the too frequent and reckless use of floss silk, the use of it in dentistry should be discarded, no virtue in it, a fancy feature in practice, nothing more, nothing less.

On the subject of root filling there is great diversity of opinion and advocacy of sundry material and modes of introduction. I have tried much that has been recommended and my conviction based upon experience is that nothing recommended is equal to beeswax and cotton fiber, it can be more easily and quickly introduced, and more perfectly precludes the possibility of ingress of fluids to nerve canal than any other material, it is "non-conducting, non-irritant and non-destructible," three very important requisites in the operation of root filling.

Capping exposed nerves is a risky business and should not be ventured often, now and then there may be a success, but so seldom compared to failures, the risk is hardly worth taking. A nerve devitalized and removed from sentient extremity of root canal, admits of promise of comfort to patient and preservation of tooth for a great while, generally speaking, patients are satisfied with and appreciate such operations, but complain heavily and justly so if effort to save by capping proves a failure.

Operative dentistry, or in other words, proper care and treatment of the mouth, teeth and soft tissues from early childhood to years of maturity, would in the course of time (a few decades) abate to a very considerable extent necessity for mechanical dentistry, and this is to be the ultimate destiny of the profession, all should hope for it and labor for it, especially in dental colleges.

All of dentistry (operative) does not consist in ability to make an ornamental display of gold in the teeth, the ability is commendable but the display is censurable and should be avoided when possible.

Decay should never advance so far as to admit of and require contour display fillings. Let us study and work to avoid that state of things. We should begin with children and let our treatment be preventive rather than restorative.

There is no justification for the wholesale extraction of teeth as is now practiced, the voice of the profession should be raised against it. It is practiced to an extent that is criminal in some cases, and should be considered a disgrace to the man or men who perpetrate the act Nature should and does revolt at such a reckless and unauthorized sacrifice of natural organs of the human body. Let us go back to the beginning, teach and be taught, to treat and preserve if possible all the natural teeth.

The first thing now with a dental student when he enters college is to learn how to prepare cavities so as to display fillings specially to catch the public eye.

The first thought and desire should be to prepare for treatment and preservation of the teeth that they may never or very rarely require fillings that would be exposed to view, then by degrees advance to preparation of and filling cavities as something necessary as a last resort rather than extract, when this course is advised and practiced in dental colleges and private offices (as must and will be in course of time) we will be acting and living on a higher plane of professional excellence, our position and claims will be more respected and our services more appreciated.

The services of dentists should be as the services of practitioners of medicine, brought within the reach of all classes in communities; poverty and misfortune should be no barrier. For this to be accomplished, there must first be reform (greatly needed) in dental college education and office practice. There must be more conservative teaching and and eclectic practice, less radicalism in both teaching and practice and a truer cultivation of spirit and disposition to do for others what they can't do for themselves, and a willingness to "live and let alive" should prevail more largely with us. The practice of high charges (fifty and hundred dollar fillings) for notoriety is censurable, unauthorized and should be discouraged and discountenanced.

There is nothing to justify such charges, no such scale of difference as to manipulative ability between fair and accomplished operators in respective localities. It creates a false impression and is demoralizing in tendency.

At our annual meetings (Conventions and Associations) it is a practice (limited I am glad to say) with some dentists to exhibit and exchange cards containing high rates of charges as if it were any evidence of manipulative excellence and superior skill. It is of no good but hurtful in effect. Like other evils, it will have its day and will as surely pass away, only to be remembered with a blush of shame. A fifty or seventy five dollar filling is of no more value as a representative means of perpetrating usefulness of teeth than a filling for which three, five or ten dollars has been charged. Why then tolerate such an unauthorized rate of charges? The profession suffers by it as would any profession tolerating such a practice. Extortion has never permanently benefitted any profession or calling in life.

Equitable fair dealing should be our basis of action in all that we do for relief of human suffering and misfortune. We should by all means avoid making false impressions upon the minds of the young men of our profession. To teach them rightly, morally and nobly by example should be our aim, and by such means elevate our profession. Impress upon their minds the importance, dignity and influence of true manhood and virtue that they may scorn to do a little thing (professionally) for notoriety or pecuniary profit, and to base every action of professional life upon a high plane, striving to be excellent in the discharge and execution of every duty and every operation with every class of patients, to ignore prejudice of any type and under all circumstances to be ambitious for knowledge and capacity to administer effectively to the wants and needs of the unfortunate and afflicted, mitigate suffering and check the ravages of disease when possible, with freedom from desire for unmerited or unenviable notoriety through trick or strategy of any sort.

And now last but not of least importance, let all of us young and old come together as one individual brotherhood, working the interest of suffering humanity, consent to subdue and control prejudice to the extent that we may be willing as members of a rising, progressive profession in our efforts in operative dentistry, consent to be truly eclectic in theory and practice, and in treating for the preservation of the natural teeth, faithfully try all things favorably recommended for filling cavities from gold down or from gold up, and as honest liberal minded true men, praise and endorse cheerfully whatever gives us good results that our professional manhood may be strengthened, our relationship one to another become more harmonizing and pleasant, thereby cultivate in the estimation of the public and our respective patrons, a higher regard and truer appreciation of the noble profession we represent and love, and from which we all realize pleasure and many of us daily subsistence.—*Southern Dental Journal*

